# Asymmetric, dynamic adaptation in prefrontal cortex during dichotic listening tasks

**DOI:** 10.1117/1.NPh.7.4.045008

**Published:** 2020-11-04

**Authors:** Jonathan A. N. Fisher, Iryna Gumenchuk, Ora S. Rogovin, Arjun G. Yodh, David R. Busch

**Affiliations:** a New York Medical College, Department of Physiology, Valhalla, New York, United States; b University of Pennsylvania, Department of Physics and Astronomy, Philadelphia, Pennsylvania, United States; c University of Texas Southwestern Medical Center, Department of Anesthesiology and Pain Management, Dallas, Texas, United States; d University of Texas Southwestern Medical Center, Department of Neurology, Dallas, Texas, United States

**Keywords:** auditory processing, diffuse correlation spectroscopy, dichotic listening, prefrontal cortex

## Abstract

**Significance:** Speech processing tasks can be used to assess the integrity and health of many functional and structural aspects of the brain. Despite the potential merits of such behavioral tests as clinical assessment tools, however, the underlying neural substrates remain relatively unclear.

**Aim:** We aimed to obtain a more in-depth portrait of hemispheric asymmetry during dichotic listening tasks at the level of the prefrontal cortex, where prior studies have reported inconsistent results.

**Approach:** To avoid central confounds that limited previous studies, we used diffuse correlation spectroscopy to optically monitor cerebral blood flow (CBF) in the dorsolateral prefrontal cortex during dichotic listening tasks in human subjects.

**Results:** We found that dichotic listening tasks elicited hemispheric asymmetries in both amplitude as well as kinetics. When listening task blocks were repeated, there was an accommodative reduction in the response amplitude of the left, but not the right hemisphere.

**Conclusions:** These heretofore unobserved trends depict a more nuanced portrait of the functional asymmetry that has been observed previously. To our knowledge, these results additionally represent the first direct measurements of CBF during a speech processing task recommended by the American Speech-Language-Hearing Association for diagnosing auditory processing disorders.

## 1 Introduction

Comprehending speech leverages neural infrastructure that spans the majority of the central nervous system. The auditory pathway alone includes both ascending and descending pathways that begin with sensory hair cells in the cochlea and ultimately project to prefrontal cortical regions. Thus, damage to or dysfunction of any aspect of the central nervous system often interferes with aspects of speech perception, such as comprehension in noisy environments.^1^^,^^2^ Behavioral tasks that probe speech processing therefore hold potential as more general neurological assessment instruments. These tasks are clinically attractive because they are easy to administer and have well-established normative values. Among such tests are dichotic listening tasks.^3^^,^^4^ Dichotic listening tasks assess an individual’s ability to retain or selectively attend to simultaneously presented sounds that differ between the ears. These tasks have been extensively explored and can uncover otherwise occult neural dysfunctions for a very wide range of conditions.^5^ For example, a common implementation for this test is for the diagnosis of auditory processing disorders.^6^^–^^9^ A more precise understanding of the kinetics of brain activity during dichotic listening tests in healthy individuals could be used to derive functional biomarkers for those and other pathologies that cannot be detected anatomically through clinical imaging.

One robust empirical characteristic of dichotic listening tests is that right-handed subjects consistently retain and repeat information presented to the right ear more accurately than the left. This phenomenon is termed a “right ear advantage” (REA).^10^^,^^11^ The left hemisphere auditory areas are dominant for language processing and thus retaining info from the left ear is an intrinsically “uphill” task. Imaging with O15 positron emission tomography (O15 PET)^12^^,^^13^ indeed demonstrates that for temporal cortical auditory areas, there is a left-biased asymmetric activation during dichotic listening. Otherwise REA has been proposed to reflect an embedded anatomical bias for information transfer, specifically through the corpus callosum,^4^^,^^14^^,^^15^ or else an active left-hemisphere priming due to attentional networks,^16^ potentially of subcortical origin.^17^^,^^18^ In fact, “split-brain” patients who have had their corpus callosum transected are not able to recall numbers presented to the left ear.^19^

At the level of prefrontal cortex, however, the left hemisphere dominance during dichotic listening tasks is not as apparent. In fact, imaging studies have found that dichotic listening tests can evoke comparatively larger activation in the right prefrontal cortex.^20^^–^^22^ Multiple rationales have been proposed for these findings. For example, this may reflect the right hemisphere devoting additional compensatory activation to retain info from the left ear given the right hemisphere’s relative disadvantage for speech and language.^23^ Alternatively, the same callosal or subcortical substrates proposed to underlie REA may be sufficiently multifunctional to evoke this greater activation.^24^ The right prefrontal cortex activity has also been proposed to reflect a more generalized, modality-neutral attentional network.^13^ Of course, given the qualitative nature of all of these models, these mechanisms are not mutually exclusive.^22^

One approach for assessing the relevance of right prefrontal cortex to more general, modality-neutral performance optimization would be to monitor the time-course of regional functional activation during repeated behavioral tasks. Repetition on any behavioral task alters performance, an outcome that involves dynamic plasticity in myriad cognitive processes such as working memory management, attention, and information processing. Even at the level of concise sensory-evoked responses, repeated stimuli elicit progressively smaller responses only in regions closely involved with execution of that task or handling of that information.^25^^,^^26^ Regions closely involved in orchestrating this process, putatively right prefrontal cortex, would be expected to exhibit dynamic activation that covaries with changes in task execution and performance.

Dynamic aspects of functional activation during dichotic listening, however, have been challenging to acquire at high temporal resolution using magnetic resonance imaging (MRI)-based modalities because of technical limitations related to scanner noise. For example, in their functional MRI (fMRI) study on dichotic listening, Jancke and Shah found that the background level of noise was 70 to 80 dB sound pressure level (SPL),^22^ even after attenuation by protective headphones. Facing the same limitations, Thomsen et al.^27^ identified activated regions but were limited in their ability to collect multiple time points during listening task blocks. In a related study by Schmithorst et al.,^28^ the signal-to-noise of fMRI measurements was low enough that sophisticated statistical methods were required to attribute regional activity to the task. Finally, scanner noise can alter the auditory pathway itself by eliciting reflexive middle-ear muscle contractions (stapedius reflex), or by activating the medial olivocochlear efferent reflex, which attenuates afferent auditory signals in response to loud sounds. Although a popular strategy is to scan in between listening trials to avoid the confound, these efferent effects (which are activated at the intensities involved in MRI scanning) can persist up to 50 s after loud sounds.^29^ In terms of other clinical modalities, PET has insufficient temporal resolution and nonimaging modalities such as EEG or magnetoencephalography do not have the requisite spatial resolution to probe regional changes in activation.

To avoid these systematic confounds, we monitored regional cerebral blood flow (CBF) during a repeated, free-recall dichotic digits listening task in healthy subjects using an optical technique, diffuse correlation spectroscopy (DCS). DCS takes advantage of the dynamic scattering of light from moving red blood cells to directly measure CBF and is particularly sensitive to flow in the cortical microvasculature due to the high absorption (and thus low probability of photon escape) in larger blood vessels.^30^ We monitored changes in the prosody of recorded verbal responses as a proxy for performance changes.

## 2 Materials and Methods

### 2.1 Participants

Thirteen healthy, right-handed subjects (nine women, four men age range 16 to 37) participated in this study, which was approved by the Institutional Review Board at New York Medical College and Westchester Medical Center. All methods were carried out in accordance with the relevant ethical guidelines and regulations of both institutions. Informed consent was obtained from all participants and/or their legal guardian/s prior to the measurement session.

### 2.2 Listening Tasks

**Fig. 1:**
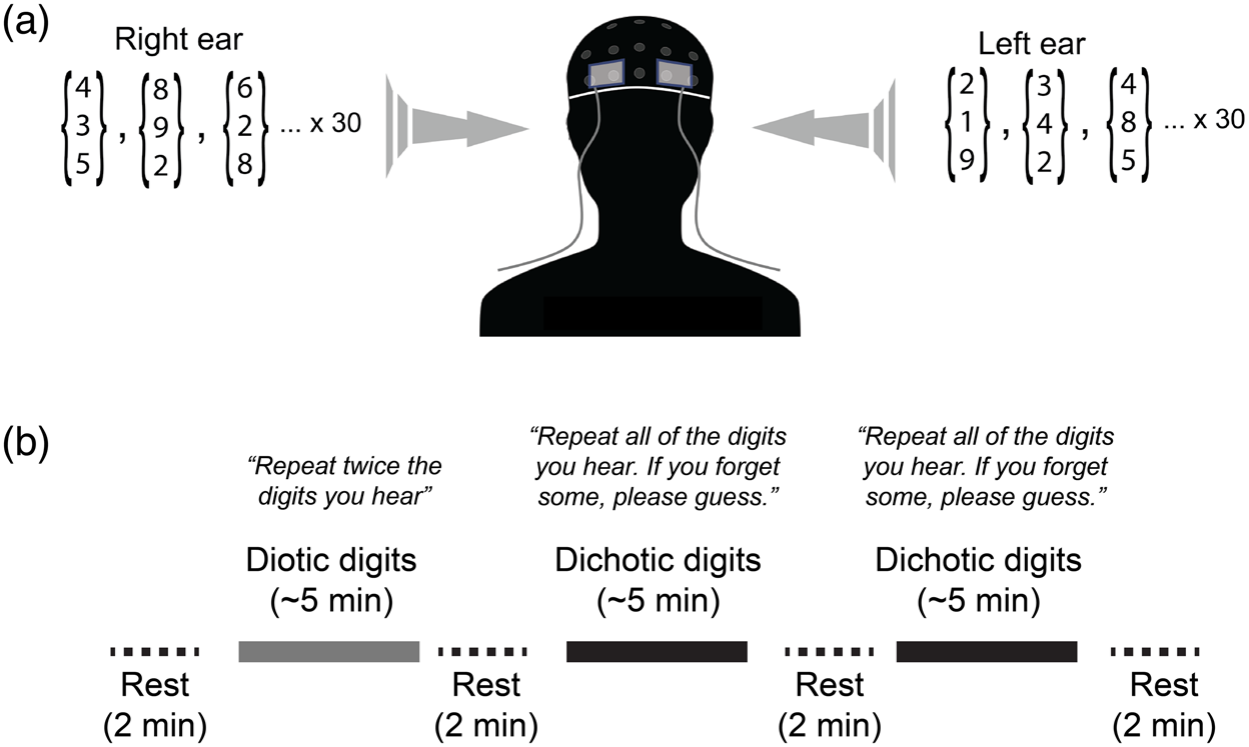
Experimental apparatus and protocol. (a) Optical probes embedded in flexible elastomer were placed at locations AF7 and AF8 (10-10 international electrode placement system) and secured under a neoprene EEG cap. The three-digit arrays depict the groups of three spoken digits that were selected randomly and delivered to each ear through calibrated insert earphones during dichotic listening tasks. (b) A block paradigm timeline indicating the timing of test blocks and rest periods. Specific paraphrased instructions are shown above each task block.

Subjects wore insert earphones (ER3-14A, Etymotic Research, Elk Grove Village, Illinois) and performed diotic and dichotic digits listening tasks. During dichotic digits task blocks (Fig. 1), each ear was presented with three sequentially spoken numbers selected randomly from 1 to 10, excluding “seven,” so that all words had only one syllable. The listening test protocol was adapted from Musiek.^31^ Each ear was presented with three different, nonrepeating numbers so that in total, a subject was presented with six different, randomly selected numbers. Spoken digits were presented as simultaneous pairs, one digit per ear, at a pace of roughly two words/s. For example, a subject might hear “two, five, four” in one ear while simultaneously hearing “eight, ten, one” in the other. Subjects were asked to verbally report the six numbers they heard, regardless of ear or order (i.e., free recall). Subjects were advised that if they could not remember one or more of the numbers, they should just guess. Subjects were asked to say the numbers clearly, yet at a normal speaking volume, and were informed that their responses were being recorded.

During diotic digits task blocks, both ears were simultaneously presented with the same numbers in unison from a single randomly selected set of three numbers so that in total only three unique numbers were heard. Speaking can introduce mechanical artifacts in both CBF and EEG measurements. To ensure that there was not an uneven influence of mechanical artifact on diotic versus dichotic listening tests, subjects were instructed during the diotic tests to twice recite the three numbers they heard, e.g., “five one two, five one two.”

Spoken numbers were selected and stimulus waveforms assembled using custom LabVIEW code (National Instruments, Austin, Texas) and output as analog waveforms through a USB-based DAQ unit (USB-6215, National Instruments). The voices used for constructing the stimulus were computer-generated male voices. Analog waveforms were amplified by a stereo power amplifier (SA1, Tucker-Davis Technologies, Alachua, Florida) and input to earphone amplifiers (ER-1, Etymotic Research, Inc.) that were coupled to insert earphones through plastic tubing. The average output at the earphones was slightly adjusted between subjects but was within 65±5  dB SPL. Subjects were asked to confirm that they could clearly and comfortably hear the sound stimuli in the study room with all equipment and cooling apparatus active.

### 2.3 Study Design

Experimental sessions lasted ∼1  h. After an initial consenting process and brief interview, during which we described the breakdown and flow of the experiment, the subject’s hearing was screened in the office with an Ambco 650A audiometer (Ambco Electronics, Tustin, California) to ensure hearing detection at 20 dB HL at 500, 1000, 2000, and 4000 Hz in both ears. Subjects were then moved to an acoustically isolated room and remained there for the rest of the experiment. After fitting the subject with an EEG cap, gelling electrodes, and positioning optical probes, subjects were instructed how to perform the diotic and dichotic listening tests. Subjects were also given a sample of what each one would sound like and how to properly respond (e.g., announce the numbers in an even, clear voice).

The experiment followed a block paradigm and consisted of the following phases: (1) initial rest (2 min), diotic digits (∼4 to 5 min), rest 2 (2 min), dichotic digits block 1 (∼4 to 5 min), rest 3 (2 min), dichotic digits block 2 (∼4 to 5 min), and rest 4 (2 min). In the repeated dichotic digits block, the protocol was the same as the first, however because numbers were randomly selected, the stimuli differed when matched trial-to-trial with the first dichotic block. Subjects were informed when blocks were starting and when they had ended. There was typically 10 to 15 s delay between the end of one block and the beginning of the next to give subjects a moment to slightly adjust seating position if they were experiencing discomfort. During the experiment, the room was nearly entirely dark, except for computer monitors which faced away from the subject. Subjects were instructed to keep their eyes open (with the exception of blinking) and to try to maintain their gaze fixated on a target (black cross on white background) ∼8  ft from their face. Subjects were instructed to maintain this relaxed fixation even during rest periods. Given that the recording sessions occurred in the dark, we paid close attention to the subjects’ wakefulness during all experimental blocks. In between blocks, we verbally checked with study participants by asking “Are you still feeling good and ready to continue?” None of the subjects fell asleep.

The total duration of experiments was ∼25  min. In pilot experiments with other subjects prior to this experiment, we found that ∼30  min, beginning with putting on the cap and probe placement, was generally the maximum time subjects could sit before experiencing some subjective level of fatigue. Subjects varied in the duration of time required for initial setup before any data was recorded, given that optical, acoustic, and electrophysiological probes were applied to the head. In an effort to minimize intersubject fatigue differences, if setup lasted longer than 10 min, rather than risk significant fatigue, we administered only a single rest/dichotic/rest measurement sequence of blocks. Thus, a diotic and repeated dichotic block were measured for 8 out of 13 subjects.

### 2.4 Measurement of Cerebral Blood Flow Using Diffuse Correlation Spectroscopy

Regional CBF was measured using DCS, which is a near-infrared interferometric technique that directly measures microvascular blood flow.^32^^,^^33^ DCS utilizes the interference pattern formed on tissue surface following illumination by a long-coherence length laser. Fluctuations in the interference pattern are related to the displacement of red blood cells in the tissue and can be utilized to compute a blood flow index (BFI). Changes in this index from baseline reflect changes in blood flow;^34^^,^^35^ DCS has been validated in multiple studies against Doppler ultrasound,^36^ fluorescent microspheres in piglets,^37^ and MRI techniques (i.e., arterial spin-labeled perfusion and phase-encoded velocity mapping).^38^^,^^39^

**Fig. 2:**
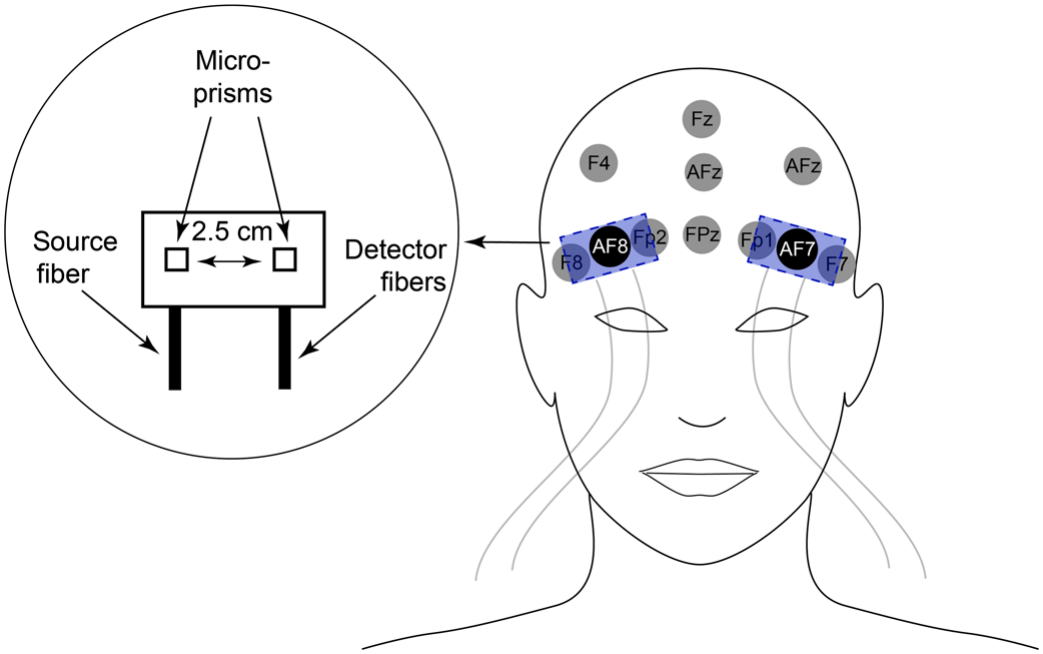
Positioning and design of optical probes. EEG recording locations in the vicinity of the probe are identified in the head image on the right. The illumination/collection from source/detector fibers was directed toward the scalp with microprisms. Source and detector prisms were separated by 2.5 cm, measured from the microprism centers. The probe was positioned on the scalp such that EEG recording locations—AF7 or AF8—were situated directly under the midpoint of the probe (equidistant from source and detector prisms).

The present study utilized a high-speed variant of DCS, capable of measurement rates of up to 50 Hz.^40^^–^^43^ Briefly, continuous wave, long coherence length lasers (785 nm; 80 mW; DL785-100-3O, 830 nm; DL830-100-3O, CrystaLaser Inc., Reno, Nevada) were used to illuminate the scalp via a prism-coupled multimode fiber (200  μm diameter, OZ Optics, Ottawa, Canada). Remitted light that traveled through the head was detected by four prism-coupled single mode fibers (780HP, 6  μm core diameter, Fiberoptic Systems Inc., Simi Valley, California; Nefern, East Granby, Connecticut) located 2.5 cm from the source. Each detector fiber directs light to a single photon-counting APD (i.e., each fiber–detector combination is independent, SPCM-AQ4C, Excelitas, Quebec, Canada). Correlation functions derived from colocated detectors were averaged. Prisms coupled to source and detector fibers were embedded in flexible pads made from pourable elastomer that were positioned on the scalp at locations corresponding to AF7 (left) and AF8 (right) in the 10-10 EEG electrode placement system (Fig. 2).^44^ The pads were slipped under a neoprene EEG cap (Enobio, Neuroelectrics, Inc., Cambridge, Massachusetts), which held them firmly, yet comfortably in place. Distances between a probe source and the contralateral detection location were >10  cm; at these distances, measurement confounds due to systematic crosstalk is negligible. In all subjects, these recording locations were ultimately >3  cm above the superior rim of the orbit, typically right at the hairline, thereby avoiding the frontal sinuses.^45^ Probe flexion due to the curvature of the head was minimal at the recording locations, and bending-induced perturbations in source-detector separation distances were within the error in geometry of the probe manufacturing.

Baseline optical properties were held fixed in the analysis (reduced scattering coefficient 10/cm; absorption coefficient 0.1/cm). Potential errors in the BFI calculation (semi-infinite homogenous model) due to a mismatch between the assumed and actual optical properties are minimized by utilizing the ratio between the BFI at each time point and that from the baseline period. We performed the fit to the normalized intensity autocorrelation function, $1.5>g_{2}(\tau )\geq 1.05$.

### 2.5 EEG Measurements

EEG was recorded wirelessly using the 10-10 international electrode placement system (Enobio 20, Neuroelectrics). The headcap was fitted with wet electrodes (NE032, Neuroelectrics) which were then filled with Sigma Gel (Parker Laboratories, Fairfield, New Jersey). Channels with an impedance <10  kΩ were included in the analysis for this paper. Analog signals were sampled at 500 Hz and transmitted to a PC via Bluetooth. Data were analyzed offline using custom Matlab (Mathworks, Natick, Massachusetts) functions as well as EEGLab.^46^ Raw EEG data were bandpass filtered from 2 to 100 Hz.

### 2.6 Analysis of Vocalizations

Subjects’ verbal responses were recorded with a monomicrophone (ACM 1b, Cyber Acoustics, Vancouver, Washington). Acoustic artifacts and experimental pauses such as the silent periods between trials and rest periods were removed from the waveform using Audacity software (Audacity Team). The data were subsequently analyzed using custom Matlab functions. We quantified the duration of responses as the time elapsed between the first and last reported numbers. Specifically, vocal response duration was measured from the first to the last point in time of each trial where the absolute magnitude of the recorded waveform exceeded that of the ambient room noise.

## 3 Results

### 3.1 Auditory Task Performance

Overall, subjects performed well on the dichotic digits task. Averaged over all subjects (n=13), in the first block, left and right ear accuracies summed over all subjects were 85±2.3% and 89±1.8%, respectively (mean±standard error of the mean). These values, including the left/right asymmetries, are consistent with normative values for three-digit dichotic listening tests in the same age group.^47^ In addition, the left/right accuracy difference was significant (P=0.01), confirming an REA.^10^^,^^11^ Sample raw data for a dichotic task section are tabulated in Table S1 and Fig. S1 in the Supplemental Materials. Among subjects who performed a second dichotic listening block (n=8), accuracies for both ears improved to 89±3.5% and 91±1.7%, and there was no significant difference in left/right ear accuracies. After experimental sessions, when asked about their general comfort level, all participants felt that the second dichotic listening block was “easier.” Most participants noted that as they progressed through the dichotic digits task blocks, they adopted the strategy of focusing on this information from one ear while relying on “passive” recall to retain and report the digits delivered to the other ear.

### 3.2 Cerebral Blood Flow

**Fig. 3:**
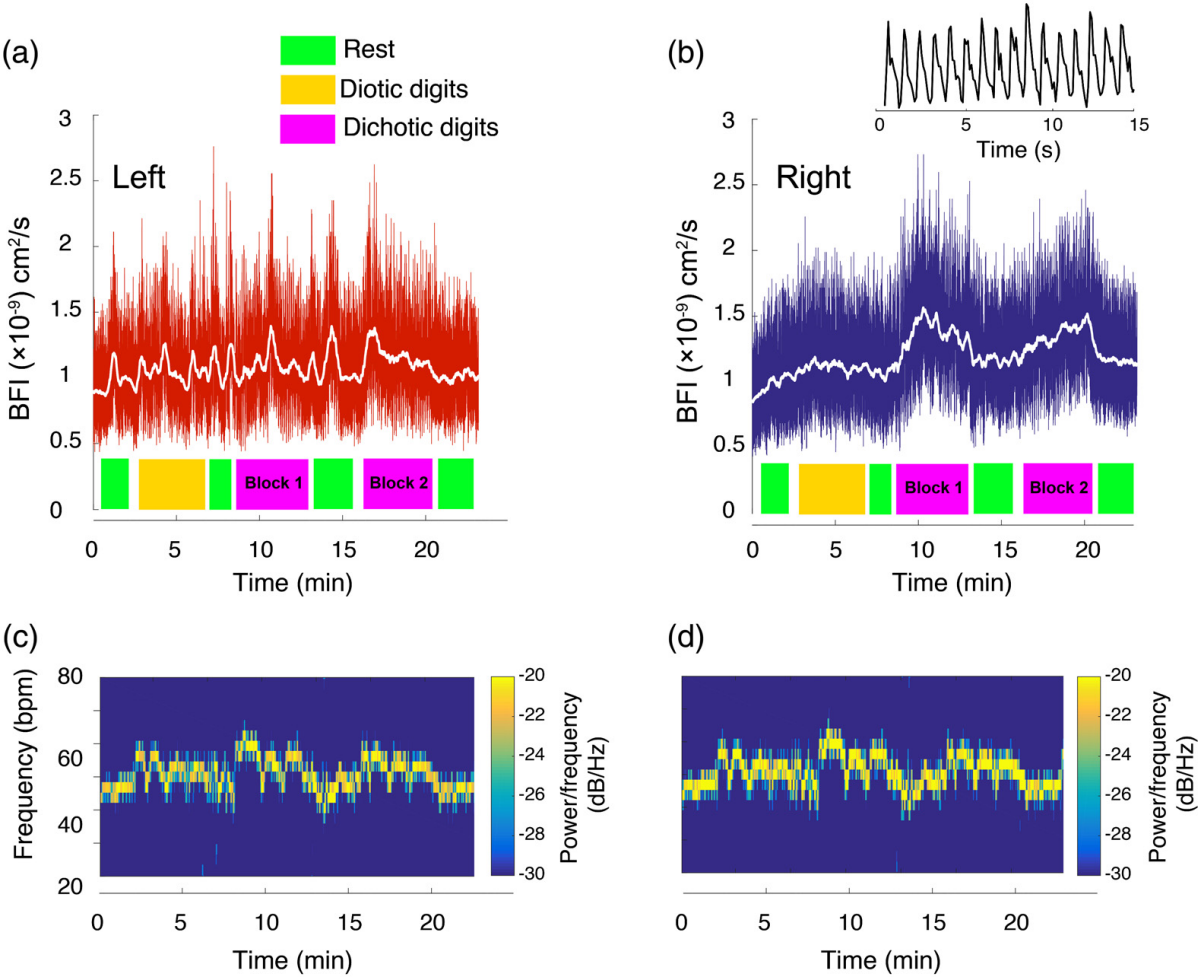
CBF in prefrontal cortex during listening tasks in one subject. (a), (b) Relative CBF measured optically at scalp positions corresponding to the (a) left and (b) right dorsolateral prefrontal cortex during a series of listening task blocks and rest periods. Values are displayed as BFI. The white trace superimposed on the data in (a) and (b) shows the panel’s same data smoothed with a 5-s moving average filter. Note that much of the “noise” is in fact flow changes due to dynamic physiology (breathing and heartbeat). The inset above (b) shows a 15-s snippet of the optical raw data showing pulsatile flow due to the cardiac cycle. Representative of the population averaged results, blood flow at the recording locations increased most significantly during dichotic listening task blocks, and the increases were larger on the right hemisphere. (c), (d) Spectrograms of the optical signals in (a) and (b), respectively, in the range of 20 to 80 Hz. These signals reflect heartrate during experiments and are symmetric, unlike the left-right asymmetry visible in the raw data plots in (a) and (b) above.

**Fig. 4:**
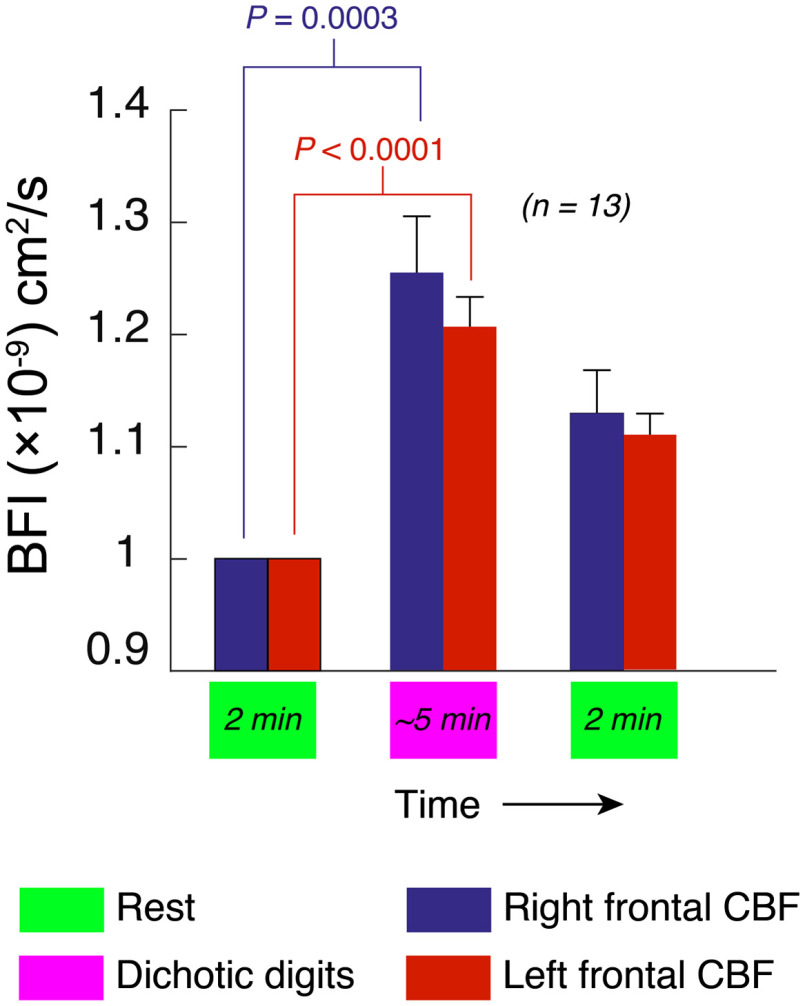
CBF activation during a diotic listening task, averaged over all subjects. Blue and red bars depict relative changes in, respectively, right and left hemispheres. The values are normalized to the first rest period. Error bars represent standard error of the mean, P-values (two-tailed Student’s t-test) are shown and “N.S.” indicates P>0.05.

As shown in Figs. 3(a) and 3(b), listening tasks induced functional changes in CBF at recording locations AF7 (left) and AF8 (right) in the 10-10 EEG electrode placement system,^44^ corresponding to the left and right dorsolateral prefrontal cortex (Brodmann areas 46L and R). As shown in Fig. 4, averaged over all subjects, dichotic digits tasks evoked statistically significant changes in blood flow relative to the preceding rest phase, 25±5% and 21±3% in the right and left hemispheres, respectively (n=13). Diotic digits tasks, which did not require subjects to parse information between the ears, also elicited asymmetric responses, albeit of lower magnitude compared with dichotic tasks (18±5% and 12±2%, right and left). Although the measurements appeared to contain an abundance of random high frequency noise, as can be seen in the inset for Fig. 3(b), the amplitude fluctuations largely reflect pulsations in blood flow due to the cardiac cycle. In fact, because the optical sampling was rapid, we were able to extract the heartrate based on a spectrogram of the optical signals [Figs. 3(c) and 3(d)]. As shown in Figs. 3(c) and 3(d), listening tasks transiently increased heartrate. However, the increases in heartrate were hemispherically symmetric and the slow temporal evolution of the 40- to 70-Hz frequency band spectral density did not match the corresponding slow trends in the unfiltered blood flow data.

**Fig. 5:**
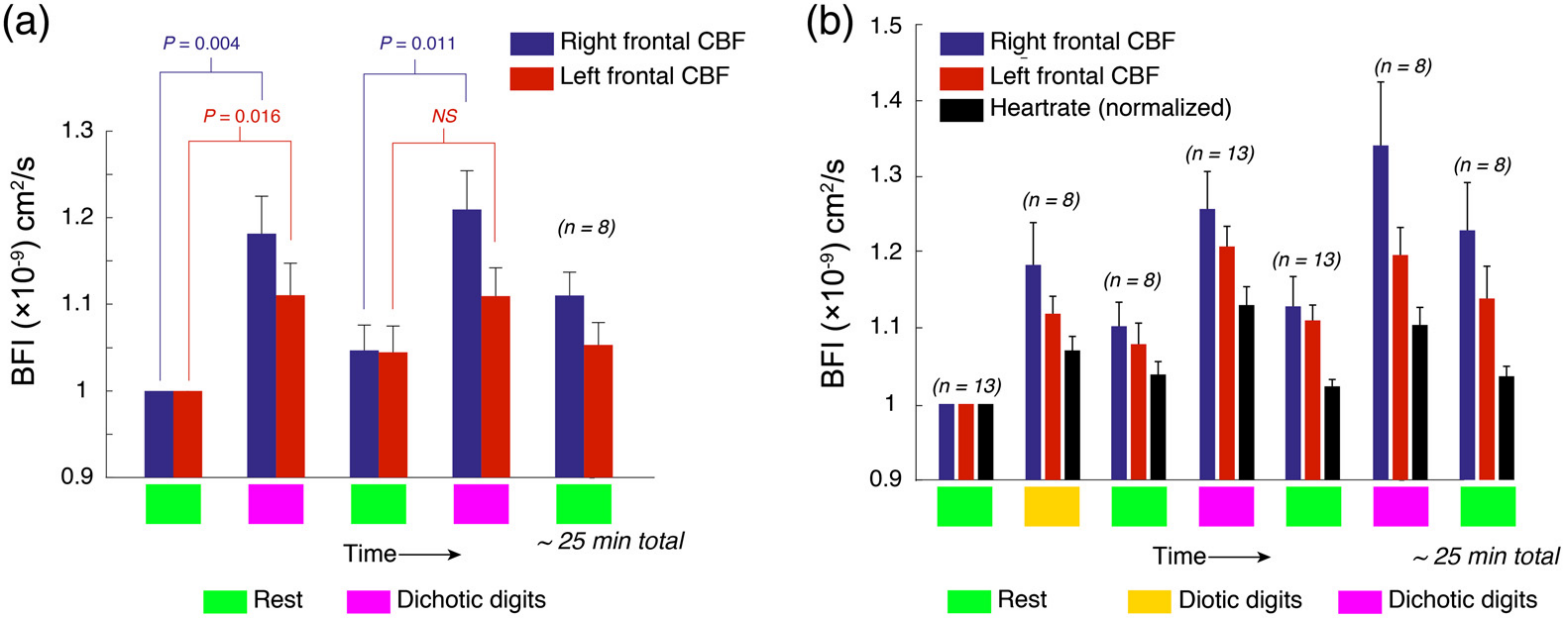
Asymmetric adaptation during repeated dichotic listening blocks. (a) In experiments where a dichotic block was repeated (8 out of 13), there was a statistically significant amplitude increase from block 1 to block 2 in the right hemisphere. In the left hemisphere, however, there was no such statistically significant difference. Values represent changes in CBF relative to the rest directly prior to the first dichotic block in this subset of eight out of experiments. (b) Hemodynamic data for all experiments, over all blocks. In addition to CBF, relative heartrate is depicted. Absolute heartrate values are tabulated in Table S2 in the Supplemental Materials. Because not all experiments featured a repeated dichotic block, the number of subjects that the data represents for each bar is indicated. Error bars represent standard error of the mean, P-values (two-tailed Student’s t-test) are shown and “N.S.” indicates P>0.05.

**Fig. 6:**
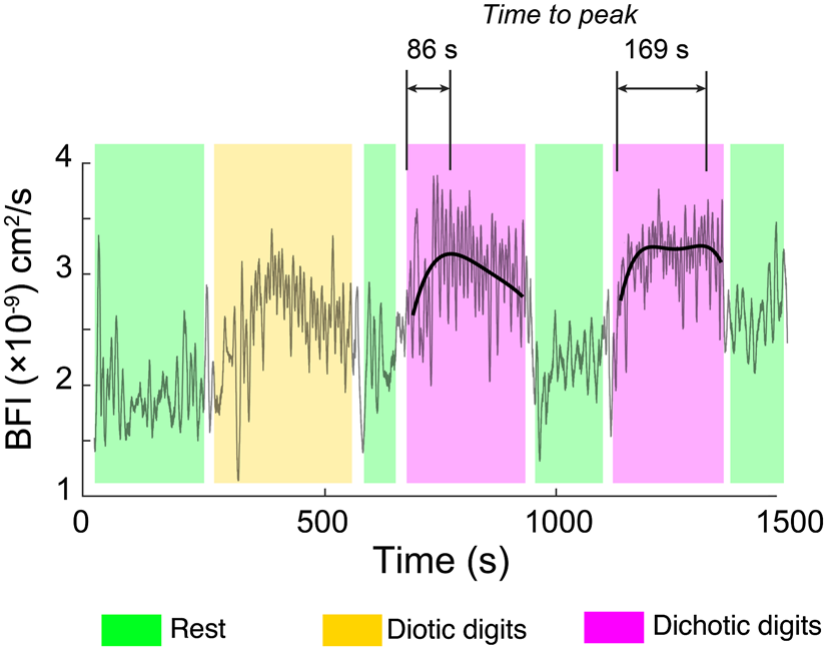
Activation kinetics in the right prefrontal cortex differs between the two dichotic listening blocks. Here, local third-order polynomial fits are superimposed over the raw optical data during the dichotic listening periods. The shaded regions indicate rest (light green), diotic (light yellow), and dichotic (light purple) blocks. The increased time-to-peak in diotic block 2 that is highlighted here is representative of the population average. No statistically significant corresponding change in kinetics was found in the left hemisphere.

In a repeated dichotic block, which was recorded in a subset of 8 out of 13 subjects, the average CBF in the right hemisphere once again increased significantly relative to the preceding rest; however, there was no statistically significant change recorded for the left hemisphere [Fig. 5(a)]. Hemodynamic data for all experiments, including changes in relative heart rate, are shown in Fig. 5(b). Data for all experimental measurements for each subject are tabulated in Table S2 in the Supplemental Materials. The kinetics of within-block changes in flow also changed from the first to the second block. Figure 6 shows one representative subject’s CBF during the two dichotic digits tasks. Superimposed on the full data trace are local polynomial fits during the dichotic listening blocks, which we used to quantify the nonlinear temporal dynamics of the response. Compared with the first dichotic block, the functional flow changes in the right hemisphere exhibited a slower increase and reached a peak value on average 77±4.3  s later than the first block. No statistically significant change in kinetics was noted in the left hemisphere, however. Subjects with low amplitude changes in CBF (three out of eight) yielded very poor fits and were not included in this analysis; however, assessed among the remaining subjects, the kinetics change was still statistically significant (P=0.028).

### 3.3 EEG

**Fig. 7:**
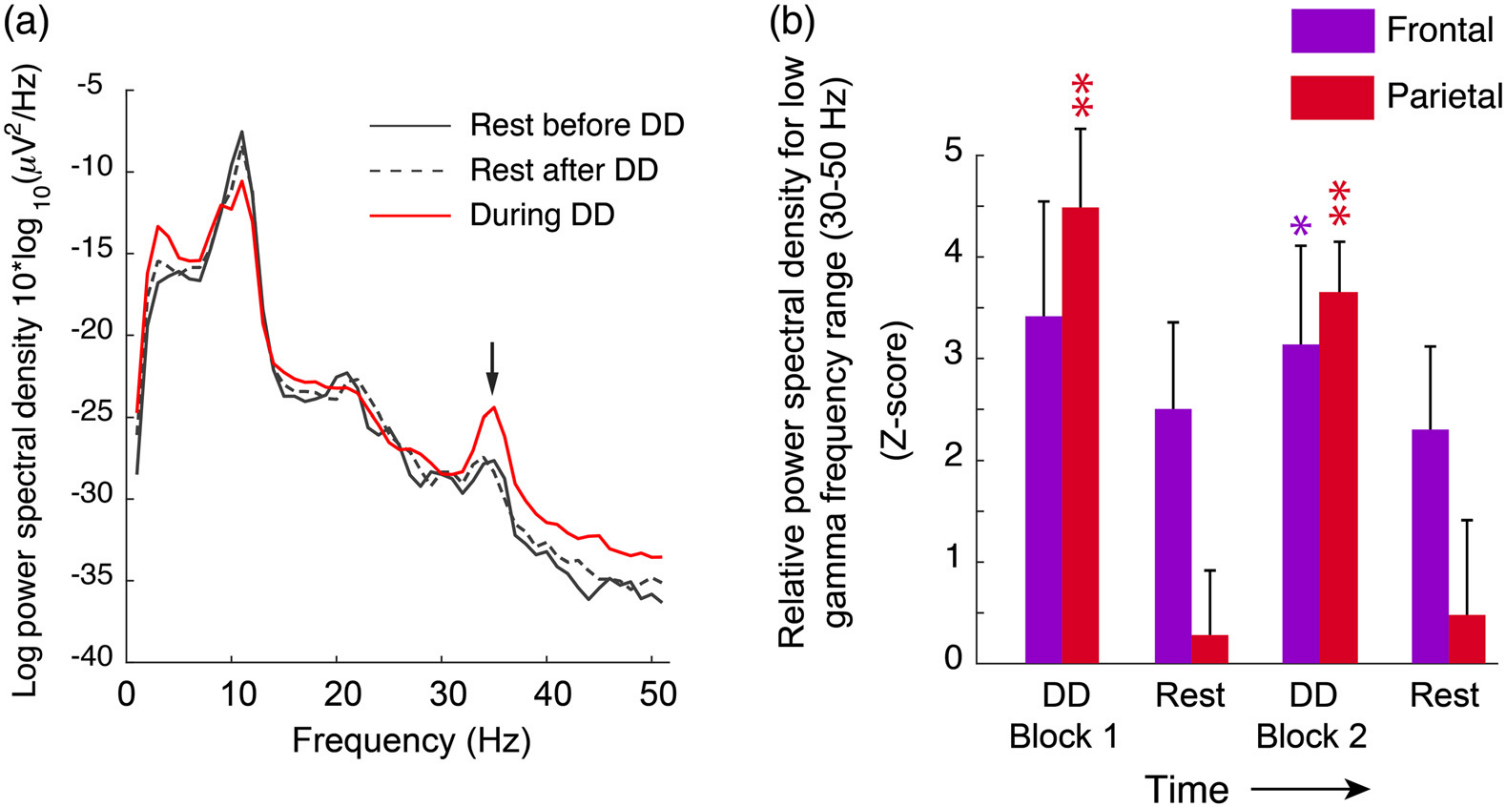
Dichotic listening increases EEG low gamma power in frontal and parietal recording locations. (a) EEG spectrum in a representative subject during dichotic listening periods (red trace) as well as rest periods before and after (solid and dotted black traces, respectively). The trace is averaged over parietal EEG recording locations. (b) Bar chart summarizing low-frequency gamma spectral power at frontal (purple) and parietal (red) recording locations during rest and dichotic digits (DD) listening periods. Because the baseline EEG power measurements varied between subjects, results are expressed as z-score, which represents the ratio of averaged spectral power during the measurement period (i.e., rest or task) to the standard deviation of preblock fluctuations. Values with z-score>2 were considered significant. Asterisks indicate statistical significance as: * = P<0.05, ** = P<0.01.

During dichotic listening task blocks, spectral features of the ongoing EEG signal at frontal and parietal recording locations displayed changes that were statistically significant. As shown in Fig. 7(a), the resting EEG spectrum featured prominent alpha (8 to 13 Hz) and beta (14 to 30 Hz) spectral features as well as a low gamma peak centered at ∼35  Hz. While diotic digits tasks, which subjects reported as subjectively “easy,” did not elicit significant changes in the EEG spectrum, dichotic task blocks were accompanied by significant changes in spectral power in the low-gamma range (30 to 50 Hz). EEG trends for subjects that received two blocks of dichotic listening tasks (8 out of 13 subjects) are shown in Fig. 7(b). Due to technical difficulties involved in maintaining audio, optical, and electrophysiological apparatuses on the head, in some subjects, the EEG electrode impedances were unstable and thus omitted from analysis. The values for all subjects and blocks are tabulated in Table S2 in the Supplemental Materials. Whereas the blood flow data for both hemispheres demonstrated a greater signal in the second dichotic block, the EEG amplitude was slightly, though not statistically significantly, greater for the first block.

### 3.4 Speech Patterns

**Fig. 8:**
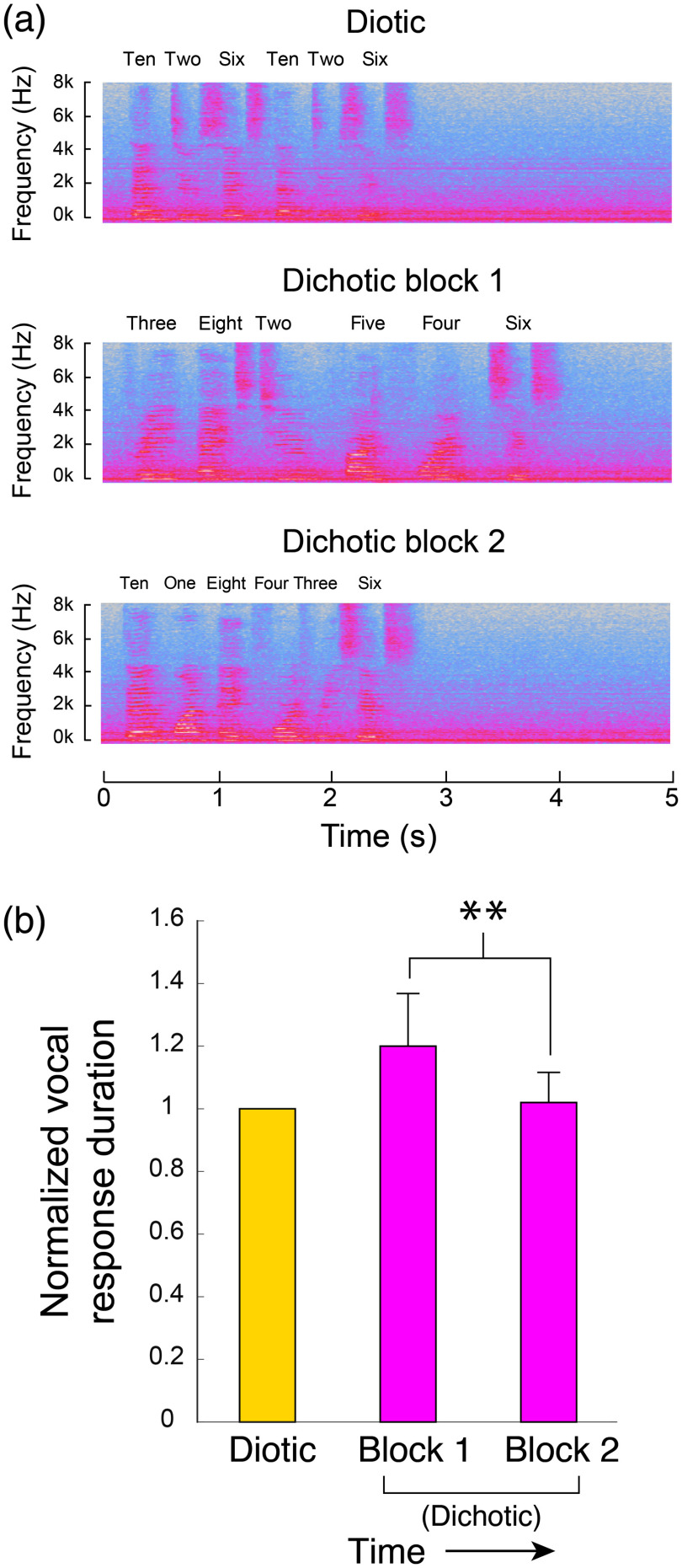
Analysis of verbal responses to listening tasks. (a) Short segments of the spectrogram of a representative subject’s verbal responses. The panels illustrate how speaking rhythm can be visualized in the time-frequency plots. The prolonged verbal response duration was most prominent in the first dichotic listening block. (b) The average total duration of the free-recall responses following auditory stimuli. Vocal response duration was defined based on the first and last time points where the total recorded audio amplitude exceeded the maximum ambient noise level in the room that was present during that test. Shown here are the average durations of a vocal response for diotic and dichotic task blocks, all normalized to the diotic response durations. The reduction in response duration visible when comparing dichotic block 1 to block 2 was significant (P=0.0079).

While the most direct metric for performance enhancement would be changes in response accuracy, the baseline accuracy was so high that we could not detect a significant change in performance across subjects, despite the fact that all subjects reported the second block being subjectively less difficult. As a metric, albeit indirect, for subjective task difficulty, we therefore assessed the speech patterns of subjects during verbal responses. When subjects were unsure or hesitant during digit recall, they repeated the numbers more slowly. We quantified this effect by measuring the total duration between the first and last spoken words. Figure 8(a) shows a representative subject’s spectrogram of the recorded sound during verbal responses. Verbal responses consisted of six spoken numbers corresponding to the six digits total (three per ear) subjects were presented with. In the diotic listening task, when both ears were presented with the same three digits, the subject was asked to repeat the three numbers twice. Compared with the responses during the diotic digits task, the total span of the verbal responses increased during the first, but not the second block of dichotic digits. These trends are quantified in Fig. 8(b), in which durations are normalized to the averaged durations during diotic tasks. These values represent the average over all subjects.

## 4 Discussion

By using optical techniques, we were able to temporally resolve the dynamics of an asymmetric activation in prefrontal cortex. These asymmetries, which included both magnitude and kinetics, likely reflect myriad underlying processes including dynamic control of attention or listening strategy, streamlining working memory, or processing efficiency, among many others. Some subjects reported that they actively explored different listening strategies during the dichotic task blocks, particularly regarding which ear to focus on or ignore. Exploratory spatial listening adjustments involved in right/left attention switching may thus have contributed to the CBF signals. Indeed, the dorsolateral prefrontal cortex receives from auditory cortex afferent information streams related to spatial localization of sounds.^48^ Future experiments in which subjects are explicitly instructed to alternate attention between the ears could more specifically guide this line of inquiry.

The EEG findings of enhanced gamma power at parietal and frontal locations during dichotic listening tasks are consistent with previous work showing that gamma spectral power is correlated with working memory engagement in both auditory and visual sensory modalities.^49^^–^^51^ The slight, albeit not statistically significant, reduction in EEG gamma power during dichotic block 2 may reflect the fact that some of the reduced subjective effort was associated with a more streamlined use of working memory. Unsurprisingly, all subjects agreed the dichotic tests were significantly more difficult than diotic, a subjective report that was supported quantitatively by the heartrate observations [Fig. 5(b)] as well as the greater regional blood flow activation during dichotic listening. This result is consistent with fMRI measurements under both diotic and dichotic listening tasks^27^ and is expected given that diotic listening does not involve a significant memory recall burden.

Importantly, we were able to use optical techniques to reproduce basic dichotic listening findings previously obtained only through fMRI. The average noise level perceived by subjects was minimal, particularly given the added hearing protection from the foam coupler tips of the insert earphones. Functional hyperemia dynamics, both onset and offset, were fast; after the final digit combination was delivered in any dichotic or diotic listening block, CBF returned to rest levels on a timescale of ∼15  s. Our ongoing measurements of regional flow exhibited a gradual upward change over the ∼30-min course of recording likely owing to subject fatigue, an issue common to most block-paradigm behavioral experiments.^52^

Because of the acoustic noise limitations associated with fMRI, diffuse optical approaches have been utilized previously by a number of groups for detecting auditory-evoked responses. Rinne et al.^53^ performed the first diffuse optical measurements of auditory tone-evoked hemodynamics at scalp regions corresponding to primary auditory cortex. Concurrently, Sato et al.^54^ utilized diffuse optical tomography to measure hemodynamics during speech processing tasks on a similar timescale as the measurements we performed. However, the authors performed measurements at temporal and parietal scalp locations, as opposed to frontal regions. Their findings of a larger activation in left hemisphere secondary auditory cortex and Wernicke’s area were consistent with the documented left hemisphere dominance for speech and language.^55^ The dichotic listening tasks, however, involved auditory targets and masking and thus technically did not match the standard clinical definitions of dichotic listening tasks.

At the level of the prefrontal cortex, some, but not all, optical imaging studies have found increased activation in the right hemisphere–and for an interestingly disparate range of tasks. Scholkmann et al.,^56^ for example, found that prefrontal cortical activation was greater in the right-hemisphere particularly during passive listening tasks. In a more targeted exploration of the foundations of auditory sensory gating, Ehlis et al.^57^^,^^58^ optically measured hemodynamics during a P50 click-suppression paradigm which has been used to explore the electrophysiological signatures of sensory gating. They observed prefrontal cortical activation that was more broadly distributed in the right hemisphere than left. On the other hand, Fallgatter et al.^59^ observed no hemispheric differences for similarly passive language processing tasks. Importantly, all of these previous studies utilize metrics of cerebral oxygenation as a proxy for blood flow, in effect, using similar assumptions as blood oxygenation level-dependent (BOLD) fMRI.

Using optical methods to probe cerebral hemodynamics has associated challenges. One source of error is related to the fact that photons that traverse the brain must also traverse the scalp and skull. As a result, the apparent blood flow measurements which are putatively of cerebral origin can also be influenced by physiological signals from the scalp and skull, for instance. As a mitigating strategy for DCS measurements, it has been shown that applying pressure to optical probes at the upper end of a subject’s comfort level significantly reduces these contributions.^34^ In the measurements reported here, while it is possible that there could be extracerebral contributions, we expect such changes would be bilateral and would not influence results regarding asymmetry of amplitude or kinetics.

As with other diffuse optical techniques, hemodynamic calculations reported by DCS are dependent on the estimated tissue optical properties used in the physical modeling. The simplicity of the apparatus and measurement configuration in this study makes it attractive from the standpoint of clinical implementation; however, because a multispectral characterization of tissue optics was not integrated, errors in estimated absorption and scattering properties can potentially have an impact on the results.^60^ In this study, the reported changes in relative CBF were derived from fitting to a semi-infinite homogeneous model, as described by Wang et al.^40^ The calculated values involve estimations of baseline optical absorption and scattering properties of the tissue, which in our case were $\mu_{a}^{0}=0.1\;{\mathrm{cm}}^{-1}$ and $\mu_{s}^{\prime 0}=10\;{\mathrm{cm}}^{-1}$. While these estimations are ubiquitous throughout the literature, they can vary from subject to subject, generally within the range of ±25%.^40^ To assess the potential impact on our reported results due to this range of variability, we reprocessed a representative subject’s data and varied the values of both $\mu_{a}^{0}$ and $\mu_{s}^{\prime 0}$ by ±25% (i.e., $\mu_{a}^{0}=0.075$, 0.1, $0.125\;{\mathrm{cm}}^{-1}$; $\mu_{s}^{\prime 0}=7.5$, 10, $12.5\;{\mathrm{cm}}^{-1}$). We explored the impact of these deviations on both the absolute BFI as well as normalized BFI (Fig. S2 in the Supplemental Materials). We found that within this range of values, the absolute BFI could vary by as +73% and −35%. However, the change in normalized BFI, which is the metric presented in this report, was at maximum 0.12%. This variability is 1 to 2 orders of magnitude smaller than the standard deviation for any of our optical measurements. It therefore seems that under- or overestimation of baseline optical properties within the range of normal variability would not significantly affect our presented results.

An additional consideration when characterizing task-related BFI changes is the potential influence of functional changes in tissue absorption and scattering properties. In terms of absorption, major physiological perturbations such as breath-holding or hyperventilation have been reported to elicit, at maximum, a ∼25% change in baseline absorption.^34^ In that study, the authors found that such a change in absorption can alter the BFI by up to 7.5% at a source–detector separation of 2.5 cm. Given that this is within the noise level of our measurements for all listening task blocks, absorption changes are unlikely to impose major scaling alterations. In terms of changes in scattering properties, the two general sources of such changes are (1) alterations in red blood cell flow properties within the cortical microvasculature and (2) optical intrinsic signals due to neural activity.^61^^,^^62^ In terms of the former, there has been substantial experimental and theoretical work characterizing the scattering aspects of blood flow and their influence on DCS (e.g., Carp et al.)^63^^,^^64^ as those effects are the signal’s physical origin. In terms of optical intrinsic signals, the scattering changes measured *in vivo* for profound physiological perturbation (e.g., middle cerebral artery occlusion stroke model) are <5%.^62^ Other causes of dramatic scattering change, e.g., edema,^65^ have little effect on the time scale of this measurement. Given these findings, it seems reasonable to assume that scattering changes would be highly unlikely to influence the apparent BFI signals.

In terms of the clinical interpretation of the optical measurements reported here, as with other clinical measurement modalities such as fMRI, systemic responses during imaging experiments involving behavioral tasks are a ubiquitous confound. Relevant to this study, listening tasks that involve digit retention, such as the dichotic listening task, have been shown to elicit increases in heart rate of 5% to 10%.^66^^,^^67^ Optical measurements, however, are perhaps more prone to reflecting such task-neutral effects because they may include extracerebral signals, as discussed above. It is apparent in the results shown in Fig. 5(b) that all listening task blocks elevate the heart rate by ∼10%, relative to rest periods. In comparison, the CBF changes, certainly the right hemisphere, are generally greater. Although our probe pressure likely reduced scalp perfusion significantly, it is indeed possible that a modality-neutral systemic response factors into our blood flow measurements of putatively cerebral origin. Global physiological changes that are not specific to the dichotic listening task, however, would likely be bilateral. In contrast, the amplitude and kinetics of the blood flow responses in the results presented here are asymmetric. Because of this, as well as the fact that fMRI measurements of the same tasks report the same asymmetries,^20^^–^^22^ it seems exceedingly unlikely that our measurements merely reflect global physiological changes.

Because the optical measurement technique we employed is relatively silent, inexpensive, and tolerant of head motions, a natural clinical implementation that our approach is relevant for is speech-language pathological assessment. The present study demonstrates the feasibility of using noninvasive optical measurements to monitor brain activity during listening task drawn from a battery of tests recommended by the American Speech-Language Hearing Association for diagnosing auditory processing disorders.^7^ While therapy for conditions such as auditory processing disorders are essential, a central limitation of current clinical assessments is that the presumed neural substrates for most conditions involving speech or language dysfunctions are not well validated. The ability to directly measure functional hemodynamics during testing and over the course of therapy therefore offers new opportunities particularly because many disorders feature similar symptoms, and in many cases the conditions are comorbid.^68^ For example, up to 70% of individuals with dyslexia have an underlying auditory processing disorder.^69^ Other neurological conditions can obscure speech-language disorders or else be comorbid. A large percentage of people diagnosed with dyslexia, for instance, are also diagnosed with attention deficit disorder with and without hyperactivity (ADD/ADHD).^69^^,^^70^ While differentially diagnosing speech-language pathological and audiological disorders is not presently realistic, these results can at very least offer insight into whether therapy is having an effect. In addition, this approach expands the technological arsenal for clinical research on the neurobiological bases for speech-language and central auditory processing disorders, much of which relies on fMRI investigation with a heavy emphasis on hemispheric asymmetries.

On a broader, societal level, the absence of quantitative metrics for speech-language pathological disorders serves to preserve current inequalities in the assessment and treatment of children. Speech-language pathological assessment and treatment procedures are generally not reimbursed by health insurance policies because it is not quantitatively demonstrable that therapy influences specific neurological functions or deficits. This leads to a significant socioeconomic bias in the referral and intervention process. For example, children in private schools are twice as likely to be diagnosed, and the vast majority of those who receive therapy are Caucasian.^71^ In addition, because many of the ASHA-recommended behavioral tests are highly dependent on language, testing tends to overdiagnose non-native English speakers and even English speakers with different regional accents.^72^

## 5 Conclusions

Assessing auditory processing disorders involves reducing the results of complex listening tasks into simple readouts such as accuracy. In efforts to establish quantitative diagnostic metrics that are more intimately related to specific neural structures, prior investigation has revealed hemispheric amplitude differences as one possible distinguishing parameter. Our findings identify additional parameters that can be used to classify normative responses, namely kinetics and asymmetric adaptation during repeated tasks. The variability of the laterality findings in the context of other studies still suggests that further investigation is needed before prefrontal cortical activity can be used to infer anatomical mechanisms underlying performance on clinical speech-language tests. Future measurements with the same apparatus and different sensory modalities may help elucidate the degree to which the asymmetries that we observed are behavioral task- or sensory modality-neutral.
