# Supplementary material for: Asymmetric, dynamic adaptation in prefrontal cortex during dichotic listening tasks
Source: Neurophotonics. 2020 Nov 4;7(4):045008. doi: 10.1117/1.NPh.7.4.045008 (PMC7641958; doi:10.1117/1.NPh.7.4.045008)
Supplement: Supplementary file 1 [file NPh_007_045008_SD001.pdf]

Subject S255	Dichotic 1								
**Trial #**	**LEFT EAR**			**# Correct**		**RIGHT EAR**			**# Correct**
0	1	2	10	3		5	6	3	3
1	3	10	2	2		3	4	2	3
2	5	6	2	2		4	1	3	3
3	2	4	1	2		3	2	6	2
4	5	4	1	1		5	3	5	3
5	5	5	2	1		5	10	3	3
6	5	4	10	2		1	5	6	2
7	5	4	10	2		2	3	1	2
8	10	4	8	2		3	6	1	3
9	1	4	3	2		2	6	10	2
10	5	2	2	3		5	4	10	2
11	5	2	10	2		5	4	2	2
12	2	10	5	2		6	4	3	2
13	2	3	4	2		2	5	3	2
14	3	2	10	3		2	4	5	1
15	5	4	5	1		3	1	3	2
16	5	3	1	2		10	3	2	2
17	2	4	6	2		3	9	6	2
18	1	4	6	2		2	3	9	2
19	1	5	4	2		10	6	3	2
20	1	4	10	3		2	2	2	2
21	3	5	2	3		6	10	4	2
22	2	1	4	2		5	2	3	3
23	2	5	2	2		10	3	6	3
24	4	1	3	3		10	2	6	2
25	4	5	2	3		10	2	3	2
26	5	1	6	3		10	9	4	2
27	5	10	1	2		4	2	2	3
28	2	4	1	2		3	10	6	3
29	2	3	6	3		3	5	10	3
			Total Correct	66				Total Correct	70
			Accurary	0.73333333				Accuracy	0.77777778

Table S1: Sample dichotic block performance log for a single subject (S55). Red highlighting indicates incorrectly reported digit.

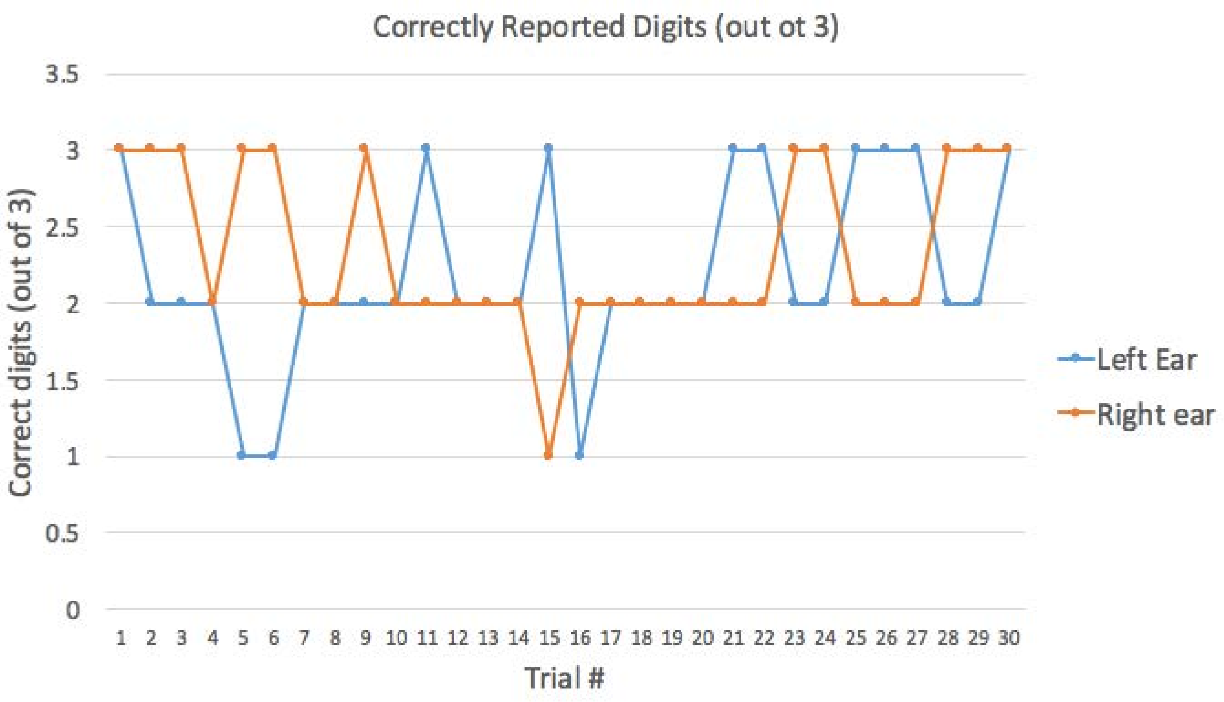

Figure S1: Plot of correctly reported digits per block for a representative study participant (raw data shown in Table S1)

Table S2

Rest 1

Subject ID (randomized)	Right / Left normalized BFI (mean ± SD)	Heartrate (bpm, mean ± SD)	Frontal / Parietal EEG gamma spectral power (30-50Hz) *z*-score *	Listening task accuracy
**S16**	(used for normalization)	76.9 ± 2.6	(rest used as reference for *z*-scores)	NA
**S46**	(used for normalization)	65.4 ± 3.4	(rest used as reference for *z*-scores)	NA
**S59**	(used for normalization)	59.6 ± 2.6	(rest used as reference for *z*-scores)	NA
**S87**	(used for normalization)	**	(rest used as reference for *z*-scores)	NA
**S44**	(used for normalization)	69.0 ± 2.1	(rest used as reference for *z*-scores)	NA
**S22**	(used for normalization)	60.7 ± 27.5	(rest used as reference for *z*-scores)	NA
**S57**	(used for normalization)	72.4 ± 2.5	(rest used as reference for *z*-scores)	NA
**S55**	(used for normalization)	80.7 ± 2.4	(rest used as reference for *z*-scores)	NA
**S80**	(used for normalization)	74.5 ± 2.3	(rest used as reference for *z*-scores)	NA
**S30**	(used for normalization)	72.3 ± 4.2	(rest used as reference for *z*-scores)	NA
**S47**	(used for normalization)	75.1 ± 2.3	(rest used as reference for *z*-scores)	NA
**S20**	(used for normalization)	64.7 ± 3.8	(rest used as reference for *z*-scores)	NA
**S83**	(used for normalization)	70.5 ± 2.1	(rest used as reference for *z*-scores)	NA
**Average over all subjects**	**1 / 1**	**69.9 ± 6.8**		

*** *z*-score for EEG is computed using the previous rest block's standard deviation**
**** Heart rate error high because the optical sampling rate was low (adjusted from subject to subject to improve S/N)**
***P* values in BFI and heart rate compare the particular block to the value measured at the first rest period (2-tailed Student's t-test). "NS" implies *P* > 0.05.**

Diotic digits

Subject ID (randomized)	Right / Left normalized BFI (mean ± SD)	Heartrate (bpm, mean ± SD)	Frontal / Parietal EEG gamma spectral power (30-50Hz) *z*-score *	Listening task accuracy
**S16**	12.0 ± 15 % / 19.6 ± 17.4%	81.0 ± 4.0	(unstable) / (unstable)	100%
**S46**	—	—	—	—
**S59**	4.9 ± 7.5% / 6.9 ± 23.1%	58.8 ± 3.7	3.9 / 2.6	100%
**S87**	—	—	—	—
**S44**	11.4 ± 5.7% / 6.1 ± 33.1%	78.2 ± 4.2	(unstable) / 1.5	100%
**S22**	—	—	—	—
**S57**	38.7 ± 10.4% / 22.2 ± 13.5%	79.5 ± 23.5	3.6 / (unstable)	100%
**S55**	3.9 ± 37.8% / 9.9 ± 4.35%	84.0 ± 14.8	0.2 / 0.6	100%
**S80**	44.9 ± 16.5% / 8.2 ± 7.6%	83.1 ± 9.0	1.1 / 3.7	100%
**S30**	18.4 ± 10.2% / 14.8 ± 8.3%	**	2.6 / 2.5	100%
**S47**	12.6 ± 14.5% / 7.2 ± 22.5%	80.3 ± 4.7	(unstable) / (unstable)	100%
**S20**	—	—	—	—
**S83**	—	—	—	—
**Average over all subjects**	**18.4 ± 15.2% / 11.8 ± 6.2%**<br>***P* = 0.011 / *P* = 0.001**	**77.8 ± 8.6**<br>***P* = 0.006**		100%

Rest 2

Subject ID (randomized)	Right / Left normalized BFI (mean ± SD)	Heartrate (bpm, mean ± SD)	Frontal / Parietal EEG gamma spectral power (30-50Hz) *z*-score *	Listening task accuracy
**S16**	6.2 ± 12.8 % / 16.2 ± 15.3%	82.4 ± 3.9	(unstable) / (unstable)	NA
**S46**	—	—	—	NA
**S59**	4.8 ± 5.3% / 5.6 ± 14.7%	60.3 ± 4.2	-0.7 / -0.6	NA
**S87**	—	—	—	NA
**S44**	6.4 ± 6.4% / 10.7 ± 35.4%	72.6 ± 4.7	(unstable) / 1.1	NA
**S22**	—	—	—	NA
**S57**	26.4 ± 6.9% / 4.3 ± 8.1&	71.0 ± 4.6	1.3 / (unstable)	NA
**S55**	2.2 ± 7.4% / 4.4 ± 22.5%	84.1 ± 2.3	-3.2 / -0.3	NA
**S80**	20.6 ± 21.9% / 5.6 ± 4.7%	83.1 ± 4.0	-0.9 / 0.8	NA
**S30**	2.2 ± 4.8% / -3.7 ± 12.9%	**	2.5 / 0.8	NA
**S47**	12.8 ± 6.7% / 20.9 ± 5.4%	75.2 ± 1.4	-2.3 / (unstable)	NA
**S20**	—	—	—	NA
**S83**	—	—	—	NA
**Average over all subjects**	**10.2 ± 9.0% / 8.0 ± 7.7%** **_P_ = 0.014 / _P_ = 0.021**	**75.5 ± 8.6** ***NS***		

Dichotic digits 1

Subject ID (randomized)	Right / Left normalized BFI (mean ± SD)	Heartrate (bpm, mean ± SD)	Frontal / Parietal EEG gamma spectral power (30-50Hz) *z*-score *	Listening task accuracy (right / left)
**S16**	23.9 ± 19.4 % / 35.1 ± 20.8%	79.7 ± 5.8	(unstable) / (unstable)	98% / 97%
**S46**	25.7 ± 4.3 % / 27.5 ± 7.4%	69.8 ± 2.9	4.2 / 4.6	84% / 76%
**S59**	9.2 ± 8.5% / 10.7 ± 29.7%	61.7 ± 7.6	5.9 / 4.9	89% / 76%
**S87**	12.0 ± 8.2% / 19.8 ± 7.9%	**	1.46 / 3.21	95% / 92%
**S44**	35.9 ± 13.1% / 8.5 ± 30.5%	82.9 ± 5.8	2.6 / 3.7	87% / 83%
**S22**	27.8 ± 10.6% / 24.8 ± 4.4%	**	2.5 / 3.6	95% / 82%
**S57**	59.2 ± 16.0% / 32.1 ± 29%	**	(unstable) / (unstable)	98% / 100%
**S55**	9.0 ± 18.5% / 7.5 ± 32%	86.1 ± 3.9	2.6 / 6.2	78% / 73%
**S80**	63.4 ± 21.2% / 20.4 ± 7.7%	88.3 ± 6.93	3.5 / 4.1	95% / 84%
**S30**	27.9 ± 13.9% / 18.3 ± 9.5%	86.1 ± 8.9	2.6 / 3.1	83% / 81%
**S47**	16.4 ± 19.1% / 24.0 ±13.5%	**	(unstable) / (unstable)	83% / 82%
**S20**	10.2 ± 10.7% / 33.0 ± 7.6%	71.5 ± 4.8	4.5 / 3.5	88% / 92%
**S83**	10.8 ± 11.3% / 9.0 ± 3.3%	86.7 ± 8.0	4.2 / 6.1	89% / 84%
**Average over all subjects**	**25.5 ± 11.3% / 20.7 ± 9.6%** **_P_ = 2.7e-4 _P_ = 5.1e-6**	**80.2 ± 9.4** **_P_ = 4.5e-4**		**89.3 ± 6.5% / 84.8 ± 8.2%** **_P_ = 0.01‡**

‡ *P*-value represents significance of right / left ear performance difference

Rest 3

Subject ID (randomized)	Right / Left normalized BFI (mean ± SD)	Heartrate (bpm, mean ± SD)	Frontal / Parietal EEG gamma spectral power (30-50Hz) *z*-score *	Listening task accuracy
**S16**	10.5 ± 8.0 % / 14.5 ± 28.0%	79.7 ±10.2	(unstable) / (unstable)	NA
**S46**	21.1 ± 4.3 % / 13.8 ± 11.6%	66.2 ± 4.7	2.6 / 0.09	NA
**S59**	14.7 ± 7.3% / 19.1 ± 20.4%	62.1 ± 3.8	3.3 / -0.1	NA
**S87**	2.3 ± 7.7% / 0.8 ± 6.3%	84.9 ± 5.9	2.0 / -2.1	NA
**S44**	16.8 ± 5.7% / 10.7 ± 29.5%	73.3 ± 3.3	3.4 / 0.08	NA
**S22**	15.6 ± 9.5% / 7.8 ± 5.0%	**	2.7 / -0.02	NA
**S57**	51.4 ± 10.1% / 20.8 ± 18.5%	69.4 ± 5.1	2.2 / (unstable)	NA
**S55**	4.7 ± 21.1% / 3.8 ± 33.4%	82.0 ± 4.2	1.7 / 1.8	NA
**S80**	21.5 ± 8.5% / 17.8 ± 7.4%	79.5 ± 2.5	2.5 / (unstable)	NA
**S30**	1.4 ± 7.9% / 2.8 ± 4.0%	**	0.4 / (unstable)	NA
**S47**	4.2 ± 11.3% / 10.0 ± 8.4%	75.2 ± 2.3	(unstable) / (unstable)	NA
**S20**	0.2 ± 4.9% / 17.3 ± 6.0%	64.9 ± 2.5	3.1 / 0.2	NA
**S83**	3.5 ± 4.8% / 4.2 ± 5.5%	72.5 ± 3.6	3.1 / 4.5	NA
**Average over all subjects**	**12.9 ± 13.8% / 11.0 ± 6.7%** ***P* = 0.038 / *P* = 0.019**	7**3.6 ± 7.4** *NS*		

Dichotic digits 2

Subject ID (randomized)	Right / Left normalized BFI (mean ± SD)	Heartrate (bpm, mean ± SD)	Frontal / Parietal EEG gamma spectral power (30-50Hz) *z*-score *	Listening task accuracy
**S16**	32.4 ± 18.5% / 40.0 ± 15.7%	83.5 ± 11.3	(unstable) / (unstable)	93% / 93%
**S46**	—	—	—	—
**S59**	11.0 ± 7.4% / 9.9 ± 18.0%	59.8 ± 4.5	4.7 / 3.1	93% / 73%
**S87**	—	—	—	—
**S44**	36.1 ± 9.8% / 17.1 ± 29.4%	79.6 ± 3.5	4.2 / 3.2	100% / 99%
**S22**	—	—	—	—
**S57**	66.5 ± 13.2% / 28.3 ± 11.5%	85.1 ± 5.3	3.9 / (unstable)	87% / 75%
**S55**	8.2 ± 19.3% / 8.5 ± 6.3%	84.8 ± 4.3	0.3 / 3.7	89% / 95%
**S80**	70.7 ± 12.0% / 19.3 ± 7.2%	82.4 ± 4.6	0.4 / 4.5	93% / 98%
**S30**	17.2 ± 8.7% / 14.6 ± 10.1%	**	4.8 / 3.1	91% / 88%
**S47**	30.1 ± 22.1% / 18.2 ± 14.4%	86.5 ± 8.5	(unstable) / (unstable)	84% / 92%
**S20**	—	—	—	—
**S83**	—	—	—	—
**Average over all subjects**	**34.0 ± 23.6% / 19.5 ± 10.3%** ***P* = 0.004 / *P* = 0.001**	**80.2 ± 9.3** ***P* = 0.004**		**91.2 ± 4.8% / 89.1 ± 9.9%** ***NS***

Rest 4

**Subject ID (randomized)**	**Right / Left normalized BFI (mean ± SD)**	**Heartrate (bpm, mean ± SD)**	**Frontal / Parietal EEG gamma spectral power (30-50Hz) *z*-score ***	**Listening task accuracy**
**S16**	14.4 ± 19.8% / 19.5 ± 37.0%	79.3 ± 4.2	(unstable) / (unstable)	NA
**S46**	—	—	—	NA
**S59**	11.9 ± 6.2% / 12.8 ± 17.0%	61.4 ± 3.9	1.9 / -1.75	NA
**S87**	—	—	—	NA
**S44**	16.6 ± 4.2% / 1.3 ± 18.9%	77.9 ± 3.6	3.7 / 0.2	NA
**S22**	—	—	—	NA
**S57**	50.6 ± 12.3% / 15.9 ± 13.5%	73.7 ± 2.7	0.06 / (unstable)	NA
**S55**	6.5 ± 14.6% / 4.9 ± 14.1%	81.2 ± 3.2	3.6 / 3.4	NA
**S80**	39.6 ± 15.9% / 17.4 ± 9.5%	76.2 ± 11.9	2.5 / 0.4	NA
**S30**	3.6 ± 8.9% / 0.4 ± 5.4%	**	4.3 / 1.5	NA
**S47**	39.1 ± 12.0% / 38.4 ± 12.0%	75.9 ± 3.5	(unstable) / (unstable)	NA
**S20**	—	—	—	NA
**S83**	—	—	—	NA
**Average over all subjects**	**22.8 ± 17.6% / 13.8 ± 12.3%**<br>***P* = 0.008 / *P* = 0.015**	**75.1 ± 6.5**<br>***NS***		

Absolute BFI

μ_a^0			
0.075	4.42 (0.56)	2.75 (0.35)	1.98 (0.25)
0.1	4.89 (0.62)	3.06 (0.39)	2.20 (0.28)
0.125	5.30 (0.67)	3.33 (0.42)	2.40 (0.31)
	7.5	10	12.5

← $\mu_s'^0$ →

Normalized BFI

μ_a^0			
0.075	1.577 (0.200)	1.578 (0.200)	1.579 (0.200)
0.1	1.579 (0.200)	1.579 (0.200)	1.578 (0.200)
0.125	1.578 (0.200)	1.579 (0.200)	1.579 (0.200)
	7.5	10	12.5

← $\mu_s'^0$ →

Figure S2: Exploration of the effect of errors in estimated baseline tissue optical properties. To assess the potential impact on our reported results due to this range of variability, we re-processed a representative subject's data (S80) using a semi-infinite homogeneous model. We explored how varying the values of both parameters by ± 25% would impact the results. The parameters we originally used for our calculations in this manuscript were $\mu_a^0 = 0.1$ cm^{-1} and $\mu_s'^0 = 10$ cm^{-1}. We assessed this matrix of 9 scenarios ($\mu_a^0 = 0.075, 0.1, 0.125$ cm^{-1}; $\mu_s'^0 = 7.5, 10, 12.5$ cm^{-1}) in terms of both absolute BFI as well as normalized BFI. BFI values are written as mean (SD).
